# Tweedie Compound Poisson Models with Covariate-Dependent Random Effects for Multilevel Semicontinuous Data

**DOI:** 10.3390/e25060863

**Published:** 2023-05-28

**Authors:** Renjun Ma, Md. Dedarul Islam, M. Tariqul Hasan, Bent Jørgensen

**Affiliations:** 1 Department of Mathematics and Statistics, University of New Brunswick, Fredericton, NB E3B 5A3, Canada; 2 Department of Statistics, University of Southern Denmark, DK-5230 Odense, Denmark

**Keywords:** best linear unbiased predictors, clustered data, random effects, repeated data, two-part models, zero-inflated data, 62J05, 62J12, 62P10

## Abstract

Multilevel semicontinuous data occur frequently in medical, environmental, insurance and financial studies. Such data are often measured with covariates at different levels; however, these data have traditionally been modelled with covariate-independent random effects. Ignoring dependence of cluster-specific random effects and cluster-specific covariates in these traditional approaches may lead to ecological fallacy and result in misleading results. In this paper, we propose Tweedie compound Poisson model with covariate-dependent random effects to analyze multilevel semicontinuous data where covariates at different levels are incorporated at relevant levels. The estimation of our models has been developed based on the orthodox best linear unbiased predictor of random effect. Explicit expressions of random effects predictors facilitate computation and interpretation of our models. Our approach is illustrated through the analysis of the basic symptoms inventory study data where 409 adolescents from 269 families were observed at varying number of times from 1 to 17 times. The performance of the proposed methodology was also examined through the simulation studies.

## 1. Introduction

Random effects models have been widely used in the analysis of multilevel data in various fields of applications. Random effects have traditionally been assumed to be covariate-independent in these models; however, such assumption may lead to ecological fallacy [1] in the interpretation of cluster or sub-cluster level covariates. The ecological fallacy occurs if the misleading inference is made about covariate effects when group level covariates are associated with individual observations directly. To account for correlation between cluster-specific random effects and cluster-specific covariates, models with covariate-dependent random effects have been introduced to handle clustered binary data [2], zero-inflated count data [3], discrete-survival data [4] and survival data [5,6]. Modeling of multilevel semicontinuous data has recently become an area of active research [7,8,9]; however, the cluster-specific and sub-cluster-specific random effects are assumed covariate-independent so far in the literature. Multilevel semicontinuous data with cluster-specific covariates are frequently observed in medical, environmental, economic and insurance studies. An example of three-level semicontinuous data with cluster or sub-cluster level covariates is the basic symptoms inventory (BSI) study data [10] where 409 adolescents from 269 independent HIV infected parents were observed at varying number of times from 1 to 17 times. The purpose of this study was to evaluate the effectiveness of an intervention. The intervention was designed based on social learning theory that focused primarily on skill building to reduce the risk from dangerous sexual practices and substance abuse [10]. At baseline, patients and adolescents were randomly assigned to the intervention (132 patients and 203 youth) or standard care (137 patients and 206 youth). The effect of intervention on adolescents was measured by the Global severity index (GSI). GSI is a composite index that based on 53 psychiatric indices which are an indicator of abnormal mental behavior [11]. Response variable of GSI scores is right-skewed with excessive number of exact zeros. Furthermore, parental, child-specific and visit-specific covariates were observed for these 409 adolescents; therefore, accounting for the covariates at their relevant levels would be more appropriate.

In this paper, we introduce a Tweedie compound Poisson model with covariate-dependent cluster-specific and sub-cluster-specific random effects for three-level semicontinuous data. Unlike two-part models [8,9], our model characterizes zero and positive components in an integral way instead of separately. In addition, the skewness can be flexibly modeled by the power index parameter of Tweedie family of compound Poisson models. Furthermore, our approach consolidates conditional and marginal modeling. The estimation of our model has been developed based on the orthodox best linear unbiased predictors (BLUP) of random effects given the data as an extension of [12]. Explicit expressions derived for BLUP predictors of random effects predictors in this paper facilitate computation and interpretation of our models. To the best of our knowledge, this is the first time a compound Poisson model is developed with covariate-dependent random effects. Tweedie compound Poisson models are often used to characterize insurance claims, rainfalls, pollutants and health data in practice.

The rest of the paper is organized as follows. After introducing our compound Poisson mixed model with covariate-dependent random effects, we discuss prediction of random effects and model estimation in Section 2 and Section 3, respectively. Our approach is illustrated through the analyses of the basic symptoms inventory data in Section 4. The simulations and conclusion are presented in Section 5 and Section 6.

## 2. Tweedie Compound Poisson Models with Covariate-Dependent Random Effects

### 2.1. The Model

The zero-inflated continuous data are usually referred to as semicontinuous data. More specifically, we only consider nonnegative semicontinuous data in this paper. Let $Y_{ijk}$ represent the semicontinuous responses recorded from the *k*th ($k=1,2,\ldots ,n_{ij}$) observation of the *j*th ($j=1,2,\ldots ,J_{i}$) sub-cluster of the *i*th (i=1,2,…,I) independent cluster. Then the response vector can be expressed as $Y={\left(Y_{111},\ldots ,Y_{11n_{11}},\ldots ,Y_{IJ1},\ldots ,Y_{IJn_{IJ}}\right)}^{\prime }$. We consider the cluster level random effect $U_{i}$ for the response of the *i*th cluster and the sub-cluster level random effect $V_{ij}$ for the response from the *j*th sub-cluster of the *i*th cluster. Let $W={\left(U^{\prime },V^{\prime }\right)}^{\prime }$ denote the vector of the random effects where $U={\left(U_{1},\ldots ,U_{i},\ldots ,U_{I}\right)}^{\prime }$ and $V={\left(V_{1}^{\prime },\ldots ,V_{j}^{\prime },\ldots ,V_{J_{I}}^{\prime }\right)}^{\prime }$ with $V_{j}={\left(V_{j1},\ldots ,V_{jj},\ldots ,V_{jJ_{I}}\right)}^{\prime }$ respectively. Our model is based on the following three assumptions:

**Assumption** **1.** *Cluster level random effects* $U_{1},\ldots ,U_{i},\ldots ,U_{I}$ *are independently distributed with gamma distribution with mean* $\mu_{i}$ *and variance* $\sigma^{2}$*. That is* $E\left(U_{i}\right)=\mu_{i}$ *and* $Var\left(U_{i}\right)=\sigma^{2}$*, where* $\mu_{i}=\exp\left(Z_{i}^{\prime }\beta_{\left(1\right)}\right)$ *with* $Z_{i}^{\prime }$ *be the cluster level covariate vector of the ith cluster.*

**Assumption** **2.** *Given the cluster level random effects* $U={\left(U_{1},\ldots ,U_{i},\ldots ,U_{I}\right)}^{\prime }$*, the sub-cluster level random effects* $V_{11},\ldots ,V_{1J_{1}},\ldots ,V_{J1},\ldots ,V_{JJ_{I}}$ *are conditionally independent following gamma distribution with mean* $\mu_{ij}U_{i}$ *and variance* $\tau^{2}{U_{i}}^{-1}$*, where* $\mu_{ij}=\exp\left(Z_{ij}^{\prime }\beta_{\left(2\right)}\right)$ *with* $Z_{ij}$ *represents sub-cluster level covariates.*

**Assumption** **3.** *Given the cluster and sub-cluster level random effect* $W={\left(U^{\prime },V^{\prime }\right)}^{\prime }$*, the responses* $Y_{ijk}$ *follow compound Poisson distribution which can be expressed as*

(1) $$\begin{array}{c} Y_{ijk}|W∼c_{p}\left(y_{ijk};\rho^{2}\right)\exp\left\{\frac{1}{\rho^{2}}\left(\frac{y_{ijk}\mu_{ijk}^{1-p}}{1-p}-\frac{\mu_{ijk}^{2-p}}{2-p}\right)\right\}\;where\;1<p<2, \end{array}$$

*where the explicit expression for* $c_{p}\left(y_{ijk};\rho^{2}\right)$ *are given by [*13*] but are immaterial in our derivation in the following sections. This Tweedie family is also known as the power-variance family since we have* $E\left(Y_{ijk}|W\right)=\mu_{ijk}V_{ij}$ *and* $var\left(Y_{ijk}|W\right)=\rho^{2}V_{ij}^{1-p}\mu_{ijk}^{q}V_{it}^{p}=\rho^{2}\mu_{ijk}^{p}V_{ij}$*. In (1),* $\mu_{ijk}=\exp\left(Z_{ijk}^{\prime }\beta_{\left(3\right)}\right)$*, where* $Z_{ijk}$ *represents observation level covariates.*

The index parameter is restricted to the range 1<p<2 for the semicontinuous response since only this sub-family of Tweedie distributions has a support of the response $Y_{ijk}$ on left-closed interval [0,+∞). In addition, gamma distributed random effects have been traditionally used in the multiplicative models for multilevel data.

### 2.2. Moment Structure

The main focus of this section is to present the moment structures of the compound Poisson mixed model with covariate-dependent random effects discussed in the previous subsection. The moments of the model can be obtained after some algebraic calculations by methods of conditioning on random effects. The unconditional mean and covariance are presented here to facilitate the parameter estimation. As $U_{i}$ independently follows gamma distribution with mean $\mu_{i}$ and variance $\sigma^{2}$, the moment structure of $U_{i}$ can be expressed as

$$E\left(U_{i}\right)=\mu_{i}\;\;\mathrm{and}\;\;\mathrm{Cov}\left[U_{s},U_{i}\right]=\delta \left(s,i\right)\sigma^{2}\mu_{i}^{2},$$

where $\delta_{\left(s,i\right)}=1$, if s=i and 0 otherwise. Following Assumption 2, the conditional mean and variance of $V_{ij}|U=u$ can be expressed as $E\left(V_{ij}|U\right)=\mu_{ij}U_{i}$ and $Var\left(V_{ij}|U\right)=\tau^{2}\mu_{ij}^{2}U_{i}$. This implies that the unconditional mean of $V_{ij}$ is

(2) $$E\left(V_{ij}\right)=\mu_{i}\mu_{ij}$$

and the covariance of $V_{st}$ and $V_{ij}$ is

(3) $$\mathrm{Cov}\left[V_{st},V_{ij}\right]=\delta \left(s,i\right)\left\{\sigma^{2}\mu_{i}^{2}\mu_{ij}\mu_{it}+\delta \left(t,j\right)\tau^{2}\mu_{i}\mu_{ij}^{2}\right\}.$$

After some extensive algebra, $\mathrm{Cov}[U_{s},Y_{ijk}]$ and $\mathrm{Cov}[V_{st},Y_{ijk}]$ can be expressed as

$$\mathrm{Cov}\left[U_{s},Y_{ijk}\right]=\delta \left(s,i\right)\sigma^{2}\mu_{i}^{2}\mu_{ij}\mu_{ijk}$$

and

$$\mathrm{Cov}\left[V_{st},Y_{ijk}\right]=\delta \left(s,i\right)\left\{\sigma^{2}\mu_{i}^{2}\mu_{ij}\mu_{it}+\delta \left(t,j\right)\tau^{2}\mu_{i}\mu_{ij}^{2}\right\}\mu_{ijk},$$

respectively. Following Assumption 3, the conditional mean and variance of $E[Y_{ijk}|W]$ can be simplified as $E\left[Y_{ijk}|W\right]=\mu_{ijk}V_{ij}$ and $\mathrm{Var}\left[Y_{ijk}|W\right]=\rho^{2}\mu_{ijk}^{p}V_{ij}$, respectively. Thus the unconditional mean of the response variable $Y_{ijk}$ can be calculated by conditioning on the vector of random effects ***W***, which can be expressed as

(4) $$E\left[Y_{ijk}\right]=\mu_{i}\mu_{ij}\mu_{ijk}=\exp\left(X_{ijk}^{\prime }\beta \right),$$

where $X_{ijk}^{\prime }=\left(Z_{i}^{\prime },Z_{ij}^{\prime },Z_{ijk}^{\prime }\right)$. Similarly the unconditional covariance $Y_{ijk}$ and $Y_{stl}$ can be simplified as

(5) $$\begin{array}{ccc} \mathrm{Cov}[Y_{stl},Y_{ijk}] & = & \delta \left(s,i\right)\left\{\sigma^{2}\mu_{i}^{2}\mu_{ij}\mu_{it}\mu_{ijk}\mu_{itl}+\delta \left(t,j\right)\left[\tau^{2}\mu_{i}\mu_{ij}^{2}\mu_{ijk}\mu_{ijl}+\delta \left(l,k\right)\rho^{2}\mu_{i}\mu_{ij}\mu_{ijk}^{p}\right]\right\}. \end{array}$$

The moments structures described above will be used to develop the estimating equations for the regression and random effect parameters in next section.

In the literature, marginal and conditional inferences refer to the transformed linearity in regression parameters under appropriate link function for marginal and conditional means, respectively [14,15,16]. Our approach consolidates both conditional and marginal modeling interpretations under one model since both the conditional mean and the marginal mean of our model are clearly linear in regression parameters under the log-link.

## 3. Estimation of Parameters

In this section, we discuss the prediction of random effects and estimation of regression and random effects parameters.

### 3.1. Best Linear Unbiased Predictors of Random Effects

As in [12], the orthodox BLUP predictors of cluster level random effects Ui can be expressed in matrix form as follows

(6) $${\hat{U}}_{i}=E\left(U_{i}\right)+\mathrm{Cov}\left(U_{i},Y_{i}\right)\mathrm{Var}{\left(Y_{i}\right)}^{-1}\left(Y_{i}-E\left(Y_{i}\right)\right).$$

Furthermore, the explicit expression of BLUP for cluster level random effects $U_{i}$ are given by

(7) $${\hat{U}}_{i}=\frac{\mu_{i}+\sigma^{2}\mu_{i}\sum_{j=1}^{J_{i}}\sum_{k=1}^{n_{ij}}w_{ij}\mu_{ijk}^{1-p}Y_{ijk}}{1+\sigma^{2}\mu_{i}\sum_{j=1}^{J_{i}}\sum_{k=1}^{n_{ij}}w_{ij}\mu_{ij}\mu_{ijk}^{2-p}},$$

where $w_{ij}=1/\left(\rho^{2}+\tau^{2}\mu_{ij}\sum_{k=1}^{n_{ij}}\mu_{ijk}^{2-p}\right)$. Similarly the BLUP predictors of the sub-cluster level random effects $V_{ij}$ can be calculated as

(8) $${\hat{V}}_{ij}=E\left(V_{ij}\right)+\mathrm{cov}\left(V_{ij},Y_{i}\right)\mathrm{var}{\left(Y_{i}\right)}^{-1}\left(Y_{i}-E\left(Y_{i}\right)\right),$$

which can be simplified as

(9) $$\begin{array}{ccc} {\hat{V}}_{ij} & = & \mu_{ij}{\hat{U}}_{i}+\tau^{2}\mu_{ij}w_{ij}\sum_{k=1}^{n_{ij}}\mu_{ijk}^{1-p}\left(Y_{ijk}-\mu_{ij}\mu_{ijk}{\hat{U}}_{i}\right)\\ & = & \rho^{2}w_{ij}\mu_{ij}{\hat{U}}_{i}+\tau^{2}\mu_{ij}w_{ij}\sum_{k=1}^{n_{ij}}\mu_{ijk}^{1-p}Y_{ijk}. \end{array}$$

The BLUP predictors for cluster and sub-cluster random effects given in Equations (7) and (9) are positive with explicit linear combinations of semicontinuous responses. These BLUP predictors provide the best prediction of the random effects among the class of all linear functions of the observed responses.

### 3.2. Estimation of Regression Parameters

In this section, we first consider the estimation for the regression parameters in the case of known random effect parameters. To do that, following [12], we differentiate the partially observed ‘joint’ log-likelihood of the Tweedie mixed model for the data and random effects with respect to $\beta ={\left(\beta_{\left(1\right)},\beta_{\left(2\right)},\beta_{\left(3\right)}\right)}^{\prime }$ and yield the partially observed ‘joint’ score function. Then we replace the random effects with their BLUP predictors to obtain an unbiased estimating function for the regression parameters β. To be specific, the score function for cluster level regression parameters can be achieved by the following score function

$$\frac{\partial \ell }{\partial \beta_{\left(1\right)}}=\sum_{i=1}^{I}z_{i}\frac{\mu_{i}^{-1}}{\sigma^{2}}\left(U_{i}-\mu_{i}\right),$$

which can be modified after including the BLUP predictors of the cluster level random effect as

(10) $$\begin{array}{ccc} \psi^{\left(1\right)}\left(\beta \right) & = & \sum_{i=1}^{I}Z_{i}\frac{\mu_{i}^{-1}\left(\beta \right)}{\sigma^{2}}\left[{\hat{U}}_{i}\left(\beta \right)-\mu_{i}\left(\beta \right)\right]=\sum_{i=1}^{I}\psi_{i}^{\left(1\right)}\left(\beta \right), \end{array}$$

where the *i*th component corresponds to the *i*th independent cluster. Similarly the sub-cluster level and observation level score functions can simplified as

(11) $$\psi^{\left(2\right)}\left(\beta \right)=\sum_{i=1}^{I}\sum_{j=1}^{J_{i}}Z_{ij}\frac{\mu_{ij}^{-1}\left(\beta \right)}{\tau^{2}}\left[{\hat{V}}_{ij}\left(\beta \right)-{\hat{U}}_{i}\left(\beta \right)\mu_{ij}\left(\beta \right)\right]=\sum_{i=1}^{I}\psi_{i}^{\left(2\right)}\left(\beta \right),$$

and

(12) $$\psi^{\left(3\right)}\left(\beta \right)=\sum_{i=1}^{I}\sum_{j=1}^{J_{i}}\sum_{k=1}^{n_{ij}}Z_{ijk}\frac{\mu_{ijk}^{1-p}\left(\beta \right)}{\rho^{2}}\left[Y_{ijk}-{\hat{V}}_{ij}\left(\beta \right)\mu_{ijk}\left(\beta \right)\right]=\sum_{i=1}^{I}\psi_{i}^{\left(3\right)}\left(\beta \right),$$

Without loss of generality, we assume that the matrix of the covariates $X={\left(Z_{i}^{\prime },Z_{ij}^{\prime },Z_{ijk}^{\prime }\right)}^{\prime }$ is of full rank. Let the global estimating function be defined by

(13) $$\psi \left(\beta \right)={\left(\psi^{\left(1\right)}{\left(\beta \right)}^{\prime },\psi^{\left(2\right)}{\left(\beta \right)}^{\prime },\psi^{\left(3\right)}{\left(\beta \right)}^{\prime }\right)}^{\prime }.$$

As in [12,17], a global matrix expression can be obtained for this ψ(β). Furthermore, a simple relationship among sensitivity matrix $S(\beta )=E_{\beta }\left\{\partial \psi \left(\beta \right)/\partial \beta^{⊤}\right\}$, variability matrix $V(\beta )=E_{\beta }\left\{\psi \left(\beta \right)\psi^{⊤}\left(\beta \right)\right\}$ and the Godambe Information matrix $J(\beta )=S(\beta ){V(\beta )}^{-1}{S(\beta )}^{⊤}$ can be obtained. Since the joint density for (Y,U) forms an exponential family in regression parameter β, the proof in [17] applies to ψ(β) as long as the score functions are linear in both U and Y. However, this linearity is clear since the score functions are in fact the right hand side of (10)–(12) with $\hat{U}\left(\beta \right)$ being replaced by U. A component-wise proof can also be found in [18]. Thus we have the following results:

**Theorem** **1.** *For Tweedie compound Poisson models with covariate-dependent random effects, the predicted global score function* ψ(β)*, sensitivity matrix* S(β) *and variability matrix* V(β) *can be expressed as follows:*

*1*. $\psi \left(\beta \right)=X^{⊤}diag\left(E(Y)\right){\mathrm{Var}}^{-1}\left(Y\right)\left(Y-E(Y)\right),$
*2*. $V\left(\beta \right)=\mathrm{Var}\left(\psi (\beta )\right)=X^{⊤}diag\left(E(Y)\right){\mathrm{Var}}^{-1}\left(Y\right)diag\left(E(Y)\right)X,$
*3*. J(β)=−S(β)=V(β).

Following [12], under mild regularity conditions, the solution of the estimating equation ψ(β)=0 is consistent and asymptotically normal with asymptotic mean β and asymptotic variance given by the inverse of the sensitivity matrix $S(\beta )=E_{\beta }\left\{\partial \psi (\beta )/\partial \beta \right\}$ as the subjects are assumed to be independent. In addition, this estimating function ψ(β)=0 is optimal in the sense that it attains the minimum asymptotic covariance for the estimator of β within the class of all linear functions of *Y* [12]. As in [12], this estimation equation ψ(β)=0 can be solved iteratively using the scoring algorithm, where the value of β is updated following

$$\beta^{*}=\beta -S^{-1}\left(\beta \right)\psi \left(\beta \right).$$

As the asymptotic variance matrix of regression parameter vector $\hat{\beta }$ is given by the inverse of the negative sensitivity matrix $-S^{-1}\left(\beta \right)$, the asymptotic variance for each component of regression parameter vector $\hat{\beta }$ is given by the corresponding diagonal element of its asymptotic variance matrix, $-S^{-1}\left(\beta \right)$; therefore, the estimated standard errors of $\hat{\beta }$ are obtained as the square root of diagonal elements of its asymptotic variance matrix, $-S^{-1}\left(\beta \right)$.

### 3.3. Estimation of Random Effects Parameters

The dispersion parameters are now assumed to be unknown. The unknown dispersion parameter is estimated using the adjusted Pearson estimator [12]. The adjusted Pearson estimate has some advantages; one of them is that the Pearson estimator is adjusted by bias correction. We may thus estimate $\sigma^{2}$ by the following adjusted Pearson estimator:

(14) $${\hat{\sigma }}^{2}=\frac{1}{I}\sum_{i=1}^{I}\frac{{\left({\hat{U}}_{i}-\mu_{i}\right)}^{2}}{\mu_{i}^{2}}+\frac{1}{I}\sum_{i=1}^{I}\frac{d(i)}{\mu_{i}^{2}},$$

where the explicit expression for d(i) can be expressed as

$$\begin{array}{ccc} d(i) & = & E{\left({\hat{U}}_{i}-U_{i}\right)}^{2}=\frac{\sigma^{2}\mu_{i}^{2}}{1+\sigma^{2}\mu_{i}\sum_{j=1}^{J_{i}}\sum_{k=1}^{n_{ij}}w_{ij}\mu_{ij}\mu_{ijk}^{2-p}}. \end{array}$$

The expression of $\tau^{2}$ is written as

(15) $$\begin{array}{ccc} {\hat{\tau }}^{2} & = & \frac{1}{I}\sum_{i=1}^{I}\frac{1}{J_{i}}\sum_{j=1}^{J_{i}}\frac{{\left({\hat{V}}_{ij}-\mu_{ij}{\hat{U}}_{i}\right)}^{2}}{\mu_{i}\mu_{ij}^{2}}+\frac{1}{I}\sum_{i=1}^{I}\frac{1}{J_{i}}\sum_{j=1}^{J_{i}}\frac{d\left(i\right)\mu_{ij}^{2}+d\left(ij\right)-2\rho^{2}d\left(i\right)w_{ij}\mu_{ij}^{2}}{\mu_{i}\mu_{ij}^{2}}, \end{array}$$

where $d\left(ij\right)=E{\left({\hat{V}}_{ij}-V_{ij}\right)}^{2}=\rho^{2}w_{ij}\left(\tau^{2}\mu_{i}\mu_{ij}^{2}+\rho^{2}d\left(i\right)w_{ij}\mu_{ij}^{2}\right)$. The expression for $\rho^{2}$ is written as

(16) $$\begin{array}{ccc} {\hat{\rho }}^{2} & = & \frac{1}{I}\sum_{i=1}^{I}\frac{1}{J_{i}}\sum_{j=1}^{J_{i}}\frac{1}{n_{ij}}\sum_{k=1}^{n_{ij}}\frac{{\left(Y_{ijk}-{\hat{V}}_{ij}\mu_{ijk}\right)}^{2}}{\mu_{i}\mu_{ij}\mu_{ijk}^{p}}+\frac{1}{I}\sum_{i=1}^{I}\frac{1}{J_{i}}\sum_{j=1}^{J_{i}}\frac{1}{n_{ij}}\sum_{k=1}^{n_{ij}}\frac{d\left(ij\right)\mu_{ijk}^{2-p}}{\mu_{i}\mu_{ij}}. \end{array}$$

As the clusters are assumed to be independent, standard estimating function theory can be applied to demonstrate the consistency and asymptotic normality for both regression and random effects parameters of our models as the number of clusters tends to infinity. In fact, this proof has actually been shown in the Chapter 5 of [18]. In addition, our estimation algorithm iterates among updating regression parameters through the scoring method, predicting random effects via BLUP predictor by (6) and (9), updating dispersion parameters through the moment estimators given in (14)–(16).

## 4. Analysis of Basic Symptoms Inventory Study Data

In this section, we apply our proposed covariate dependent semicontinuous model to the BSI study data.

### 4.1. Model Specification

In the BSI study, 409 adolescents from 269 independent HIV infected parents were followed over time between 1995 and 2002. Adolescents were observed at varying number of times from 1 to 17 times. Therefore, the data are of a hierarchical structure with observations nested within adolescents and adolescents further nested by their parents. The response of interest is the measurement of global severity index (GSI) scores with covariates at observation, adolescent and parent levels. A complete list of these variables with descriptions is given in Table 1.

**Figure 1:**
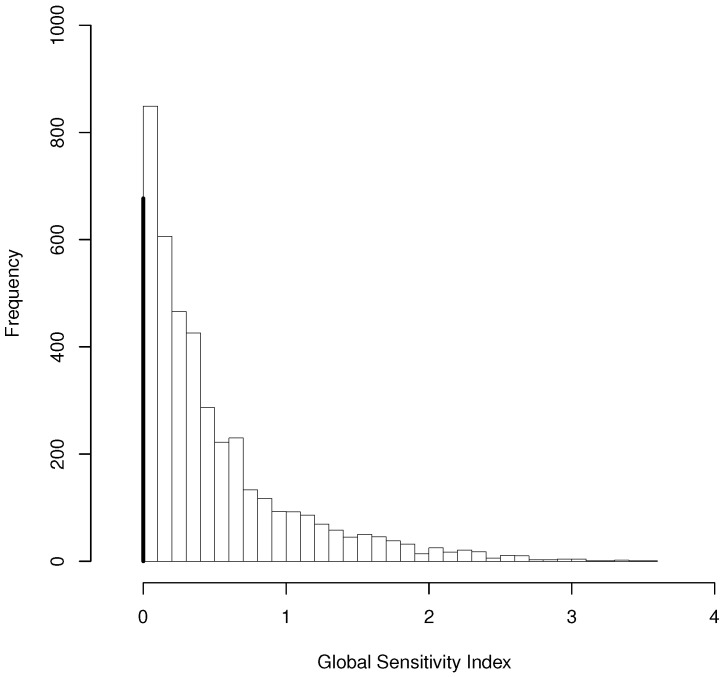
Histogram for the Global severity index (GSI) scores of Basic symptoms inventory (BSI) Data. The bold bar on the left of the histogram indicates proportion of exact zeros.

The BSI data were analyzed in [19] as longitudinal normal data ignoring parental level; however, there is a large proportion of exact zeros in the GSI response as shown in Figure 1; therefore, a three level model for semicontinuous data is more appropriate.

Let $Y_{ijk}$ represent the GSI for observation *k* from the adolescent *j* of the *i*th parent. The random effects for parent *i* and adolescent *j* of parent *i* are denoted by $U_{i}$ and $V_{ij}$, respectively. The three-level Tweedie models with covariate-dependent random effects (TMCDRE) is then specified as follows.

(17) $$\begin{array}{ccc} Y_{ijk}|V=v & ∼ & {Tw}_{p}\left(\mu_{ijk}v_{ij},\rho^{2}{v_{ij}}^{1-p}\right), \end{array}$$

with $\mu_{ijk}=\exp\left(z_{ijk}^{⊤}\beta_{\left(3\right)}\right)$. In (17), $z_{ijk}$ represents observation level covariates and $V_{ij}|U_{*}=u_{*}$ follows

(18) $$\begin{array}{ccc} V_{ij}|U_{*}=u_{*} & ∼ & \mathrm{Gamma}(\mu_{ij}u_{i},\tau^{2}{u_{i}}^{-1}), \end{array}$$

where $\mu_{ij}=\exp\left(z_{ij}^{⊤}\beta_{\left(2\right)}\right)$ with $z_{ij}$ represents sub-cluster level covariates. In (18), cluster level random effects $U_{i}$ are assumed to be independent with

(19) $$U_{i}\;\;∼\;\;\mathrm{Gamma}\left(\mu_{i},\sigma^{2}\right),$$

where $\mu_{i}=\exp\left(z_{i}^{⊤}\beta_{\left(1\right)}\right)$ with $z_{i}$ represents cluster level covariates.

### 4.2. Analysis Results

First, we obtained a maximum likelihood estimate $\hat{p}$ from Tweedie’s compound Poisson model using the R package “tweedie” Version 2.07 (Dunn, 2010) in the presence of the previous described covariates. The estimated index parameters *p* was 1.55. For p=1.55, we applied our approach to estimate regression and random effect parameters based on the model specified above. The regression and random effect parameters are presented in Table 2. To compare our proposed model with Tweedie compound Poisson models with covariate-independent random effects, we also presented analysis results based on the conventional Tweedie mixed model (CTMM) in Table 2.

Our results in Table 2 show that the effects of covariates at observation level on the GSI score did not change materially in terms of direction, significance and magnitude. Second, all covariates at adolescent level had positive effects on the GSI score with race factor clearly insignificant and gender factor highly significant. The interesting phenomenon is that effect of baseline age on the GSI score changed from significant to insignificant at the significance level of 0.05 when all covariates were considered at their relevant levels. Third, again the effects of covariates at parent level on the GSI score did not change materially in terms of direction, significance and magnitude. Our results demonstrate that analysis results may change materially when all covariates were associated at their relevant levels. We have not observed any dramatic change in this analysis. This might partly be explained by the nature of involved covariates in this study since no covariates on health status were available. Another interesting phenomenon is that the variation at the adolescent level was clearly shifted to that at the parent level when all covariates were associated at their relevant levels. A finding of practical importance is that the intervention was not effective whether covariates were associated at their relevant levels in the analysis or not.

## 5. Simulation Studies

In this section, we compare the performance of the proposed models (TMCDRE) with the conventional Tweedie mixed model (CTMM) through simulation studies to assess the effects of associating all covariates were considered at their relevant levels. To replicate data in realistic conditions, we simulated data based on analysis results given in Table 2 with respective arrangements of covariates under the TMCDRE and CTMM. The estimated regression parameters (${\hat{\beta }}_{}$) and random effect parameters ($\sigma^{2}$, $\tau^{2}$, $\rho^{2}$) under the TMCDRE and CTMM in Table 2 were taken as true values for the respective models. The simulation study under the TMCDRE and CTMM were done separately in Section 5.1 and Section 5.2.

### 5.1. Simulating Data from the Tweedie Compound Poisson Model with Covariate-Dependent Random Effects

In this section, we first generated clustered semicontinuous data from the TMCDRE model in Table 2. We then analysed the simulaed based on the TMCDRE and CTMM models. Our objective here was to compare the performance of TMCDRE and CTMM when the true data were generated from TMCDRE. The data generation procedure is given below.

- We first generate 269 samples ($u_{1},\ldots ,u_{269}$) using $\mathrm{Gamma}(\mu_{i},\sigma^{2})$ where,
  $$\mu_{i}=\exp\left(\beta_{7}\mathrm{Treatment}+\beta_{8}\mathrm{Parent}\;\mathrm{Gender}+\beta_{9}\mathrm{Parent}\;\mathrm{age}\right)$$
- In the second step, we generate $n_{i}$ samples ($v_{i1},\ldots ,v_{in_{i}}$ and $n_{i}$ varies from 1 to 5) for each $u_{i}$ using $\mathrm{Gamma}(\mu_{ij}u_{i},\tau^{2}{u_{i}}^{-1})$, where
  $$\mu_{ij}=\exp\left(\beta_{4}\mathrm{Base}\;\mathrm{age}+\beta_{8}\mathrm{Hispanic}+\beta_{7}\mathrm{Gender}\right).$$
- Finally, we generate $n_{ij}$ samples ($Y_{ij1},\ldots ,Y_{ijn_{ij}}$ and $n_{ij}$ varies from 1 to 17) for each $v_{ij}$ using $\left(v_{ij}{Tw}_{p}\left(\mu_{ijk},\frac{\rho^{2}}{v_{ij}}\right)\right)$, where
  $$\mu_{ijk}=\exp\left(\beta_{0}+\beta_{1}\mathrm{True}\;\mathrm{month}+\beta_{2}\mathrm{Spring}+\beta_{3}\mathrm{Summer}\right).$$

We carried out 400 simulation runs using the procedure discussed above. Estimated bias, simulated standard errors (Sim SE) and estimated standard errors (Est SE) of the regression and random effect parameters using TMCDRE and CTMM techniques are presented in Table 3. Parameter estimates of cluster level covariates are less biased for TMCDRE than for CTMM. Parameter estimate of the sub-cluster level continuous covariate Adolescent base age is less biased for TMCDRE than for CTMM. TMCDRE produced less biased estimates for observation level binary covariates Spring and Summer than CTMM. All dispersion parameters are overestimated by the TMCDRE, whereas only $\sigma^{2}$ and $\tau^{2}$ are overestimated by CTMM. The simulation study shows that $\rho^{2}$ is underestimated by the CTMM approach. Dispersion parameters are more biased for CTMM than for TMCDRE.

Table 3 shows the Sim SE and Est SE. Clearly, differences between Sim SE and Est SE of parameter estimates of observation level covariates (True month, Spring and Summer) are smaller for TMCDRE technique than those from the CTMM technique. Differences between Sim SE and Est SE for regression parameter estimates of all sub-cluster level covariates (Adolescents base age, Hispanic, Gender) and cluster level covariates (Treatment and Parent base age) are less for the TMCDRE than for CTMM. The only exception is with the covariate Gender.Pr. As expected, TMCDRE performed better than for CTMM when data were simulted from the TMCDRE model.

### 5.2. Simulating Data from the Tweedie Compound Poisson Model with Covariate-Independent Random Effects

In this section, we first generate clustered semicontinuous data using the conventional Tweedie Mixed Model (CTMM) method. Then, we analyze these simulated data by using TMCDRE and CTMM methods. Our objective is to compare TMCDRE and CTMM’s performances when the true data were generated based on CTMM. The data generation technique is discussed below:

- We will generate 269 samples ($u_{1},\ldots ,u_{269}$) using $\mathrm{Gamma}(1,\sigma^{2})$.
- In the second step, we will generate $n_{i}$ samples ($v_{i1},\ldots ,v_{in_{i}}$ and $n_{i}$ varies from 1 to 5) for each $u_{i}$ using $\mathrm{Gamma}(u_{i},\tau^{2}{u_{i}}^{-1})$.
- Finally, we will generate $n_{ij}$ samples ($Y_{ij1},\ldots ,Y_{ijn_{ij}}$ and $n_{ij}$ varies from 1 to 17) for each $v_{ij}$ using $\left(v_{ij}{Tw}_{p}\left(\mu_{ijk},\frac{\rho^{2}}{v_{ij}}\right)\right)$, where
  $$\mu_{ijk}=\exp\left(\beta^{T}X\right).$$

The fixed effect and random effect parameter estimates in Table 2 using CTMM technique ($\hat{\beta }=(-1.15,-0.047,0.086,-0.020,0.044,0.122,$0.401,0.0149,0.230,−0.019), $\sigma^{2}=0.10$, $\tau^{2}=0.59$, and $\rho^{2}=0.53$) are used as true parameter values. Similar to Section 5.1, we carried out 400 simulation runs using the procedure discussed above. Estimated bias, simulated stadard errors (Sim SE) and estimated standard errors (Est SE) of the regression and random effect parameters using TMCDRE and CTMM techniques presented in Table 4.

Our simulated results indicate that although data are generated from CTMM, parameter estimates from TMCDRE for observation level continuous covariate True month is less biased than the corresponding parameter estimate from CTMM. TMCDRE parameter estimate for the cluster level binary covariate Parent gender is also less biased than the corresponding parameter estimate from CTMM. CTMM gives less biased estimates for all other covariates than the TMCDRE. Dispersion parameters $\sigma^{2}$ and $\rho^{2}$ are overestimated by both conventional and covariate-dependent approach, whereas $\tau^{2}$ is underestimated by CTMM and TMCDRE. Dispersion parameters are more biased for TMCDRE than those from CTMM are.

Table 4 also indicates that the differences between Sim SE and Est SE for parameter estimate of observation level binary covariate (Spring) are smaller for TMCDRE than the CTMM. One important feature of TMCDRE is that covariates are separated in their corresponding level. The simulation study shows that the differences between Sim SE and Est SE of parameter estimates corresponding to binary covariates (Hispanic, Gender) in sub-cluster level are smaller for TMCDRE than for CTMM. Differences between Sim SE and Est SE for all other covariate are less for CTMM than TMCDRE. In other words, when data were generated from the CTMM, CTMM performed better than TMCDRE in general; however, TMCDRE still produced better estimates for some covariates than the CTMM.

## 6. Conclusions

In this paper, we have introduced a three-level Tweedie compound Poisson model with covariate-dependent random effects for semicontinuous data. As random effects at different levels are likely to be associated with the covariates at relevant levels, this new model enables us to account for such association in our analysis. Our analysis of BSI data demonstrate that analysis results may change materially when all covariates were associated at their relevant levels. Our simulation study has shown that Tweedie compound Poisson model with covariate-dependent random effects performs better than Tweedie compound Poisson model with covariate-independent random effects when random effects at different levels are associated with the covariates at relevant levels. Although we also compared performances between models with covariate-dependent and covariate-independent random effects when random effects at different levels are not associated with the covariates at relevant levels, such no association situation is far less likely in practice. Therefore, Tweedie compound Poisson model with covariate-dependent random effects is more appropriate for analysis of multilevel semicontinuous data with covariates at different levels.

Tweedie compound Poisson model with covariate-dependent random effects becomes far more complex than Tweedie compound Poisson model with covariate-independent random effects; however, we still obtained explicit expression for random effects predictors at different levels. These explicit expressions may facilitate interpretation of clustering effects at cluster and sub-cluster levels.

Models with covariate-dependent random effects have been introduced to handle multilevel binary, zero-inflated and survival data in the literature. Our model for multilevel semicontinuous data has enriched this class of models. Furthermore, Tweedie models with covariate-dependent random effects have also been developed in [18,20] for multilevel continuous and count data.

## Figures and Tables

**Table 1:** Variable description for the BSI data.

Variable Type	Name	Description
Response	GSI	BSI global severity index.
Explanatory	Cluster level	Parent level
	Treatment	Intervention or not.
	Gender.Par	1 if Parent is female or 0 otherwise.
	Age.Par	Parent’s baseline age.
	Sub-cluster level	Adolescent level
	Age.Adol	Adolescent’s baseline age.
	Race.Adol	1 if Adolescent is Hispanic or 0 otherwise.
	Gender.Adol	1 if Adolescent is female or 0 otherwise.
	Observation level	
	Months	Number of months adolescent in the study.
	Spring	Spring season.
	Summer	Summer season.

**Table 2:** Parameter estimates for the BSI data based on CTMM and TMCDRE.

Levels	Covariates	TMM			TMCDRE		
		Estimates	St. Errors	*p*-Value	Estimates	St. Errors	*p*-Value
Observation	Intercept	−1.1574	0.4449	0.0093	−1.2114	0.4515	0.0074
	Months	−0.0473	0.0072	0.0000	−0.0503	0.0071	0.0000
	Spring	0.0866	0.0325	0.0061	0.0880	0.0316	0.0045
	Summer	−0.0204	0.0338	0.5353	−0.0173	0.0326	0.5892
Adolescent	Age.Ad	0.0444	0.0221	0.0455	0.0403	0.0221	0.0719
	Race.Ad	0.1226	0.0908	0.1802	0.1148	0.0908	0.2077
	Gender.Ad	0.4017	0.0863	0.0001	0.4058	0.0863	0.0000
Parent	Treatment	0.0149	0.0916	0.8729	0.0110	0.0918	0.9045
	Gender.Pr	0.2305	0.1240	0.0643	0.2310	0.1326	0.0819
	Age.Pr	−0.0199	0.0092	0.0308	−0.0165	0.0095	0.0854
	$\sigma^{2}$	0.1007			0.1050		
	$\tau^{2}$	0.5921			0.3844		
	$\rho^{2}$	0.5316			0.6794		

**Table 3:** Summary statistics for 400 simulations from Tweedie model with covariate-dependent random effects.

Levels	Covariates	True Value	CTMM			TMCDRE		
			Bias	Sim. SE ^*a*^	Est. SE ^*b*^	Bias	Sim. SE	Est. SE
Observation	Intercept ($\beta_{0}$)	−1.2114	−0.0146	0.4463	0.4401	−0.0238	0.4354	0.4439
	Months ($\beta_{1}$)	−0.0503	−0.0004	0.0075	0.0081	0.0006	0.0073	0.0086
	Spring ($\beta_{2}$)	0.0880	−0.0015	0.0303	0.0317	−0.0008	0.0296	0.0309
	Summer ($\beta_{3}$)	−0.0173	−0.0004	0.0318	0.0312	0.0002	0.0317	0.0312
Sub-cluster	Age.Ad ($\beta_{4}$)	0.0403	0.0012	0.0234	0.0218	0.0010	0.0233	0.0219
	Race.Ad ($\beta_{5}$)	0.1148	0.0007	0.0906	0.0899	0.0009	0.0897	0.0895
	Gender.Ad ($\beta_{6}$)	0.4058	0.0062	0.0887	0.0852	0.0062	0.0871	0.0845
Cluster	Treatment ($\beta_{7}$)	0.0110	0.0081	0.0920	0.0909	0.0076	0.0906	0.0906
	Gender.Pr ($\beta_{8}$)	0.2310	0.0093	0.1247	0.1229	0.0090	0.1232	0.1254
	Age.Pr ($\beta_{9}$)	−0.0165	−0.0007	0.0100	0.0091	−0.0004	0.0098	0.0094
	$\sigma^{2}$	0.1050	0.0018	0.0561		0.0034	0.0551	
	$\tau^{2}$	0.3844	0.1871	0.1000		0.0117	0.1170	
	$\rho^{2}$	0.6794	−0.1471	0.0718		0.0270	0.1201	

^*a*^: Sim. SE, standard error of estimates over 400 simulations. ^*b*^: Est. SE, average of 400 estimated standard errors.

**Table 4:** Summary statistics for 400 simulations from Conventional Tweedie mixed model.

Levels	Covariates	True Value	CTMM			TMCDRE		
			Bias	Sim. SE ^*a*^	Est. SE ^*b*^	Bias	Sim. SE	Est. SE
Observation	Intercept ($\beta_{0}$)	−1.1574	−0.0053	0.4170	0.4373	−0.0001	0.4193	0.4455
	Months ($\beta_{1}$)	−0.0473	−0.0001	0.0071	0.0071	0.0001	0.0072	0.0070
	Spring ($\beta_{2}$)	0.0866	−0.0002	0.0332	0.0318	−0.0004	0.0331	0.0320
	Summer ($\beta_{3}$)	−0.0204	−0.0003	0.0342	0.0326	−0.0004	0.0342	0.0324
Sub-cluster	Age.Ad ($\beta_{4}$)	0.0444	−0.0014	0.0205	0.0217	−0.0016	0.0205	0.0219
	Race.Ad ($\beta_{5}$)	0.1226	−0.0029	0.0836	0.0893	−0.0031	0.0848	0.0897
	Gender.Ad ($\beta_{6}$)	0.4017	0.0020	0.0833	0.0846	0.0028	0.0840	0.0845
Cluster	Treatment ($\beta_{7}$)	0.0149	0.0010	0.0892	0.0902	0.0014	0.0891	0.0908
	Gender.Pr ($\beta_{8}$)	0.2305	−0.0052	0.0091	0.0090	−0.0047	0.0091	0.0094
	Age.Pr ($\beta_{9}$)	−0.0199	−0.0003	0.1240	0.1221	−0.0005	0.1232	0.1262
	$\sigma^{2}$	0.1007	0.0036	0.0544		0.0104	0.0552	
	$\tau^{2}$	0.5921	−0.0294	0.1029		−0.2436	0.1397	
	$\rho^{2}$	0.5316	0.0030	0.0305		−0.1576	0.1001	

^*a*^: Sim. SE, standard error of estimates over 400 simulations. ^*b*^: Est. SE, average of 400 estimated standard errors.

## Data Availability

The data are available from https://robweiss.faculty.biostat.ucla.edu/book-data-sets (accessed on 14 March 2023).
